# Longitudinal Metabolomics Profiling of Parkinson’s Disease-Related α-Synuclein A53T Transgenic Mice

**DOI:** 10.1371/journal.pone.0136612

**Published:** 2015-08-28

**Authors:** Xi Chen, Chengsong Xie, Lixin Sun, Jinhui Ding, Huaibin Cai

**Affiliations:** 1 Transgenics Section, Laboratory of Neurogenetics, National Institute on Aging, National Institutes of Health, Bethesda, Maryland, 20892, United States of America; 2 Bioinformatics Core, Laboratory of Neurogenetics, National Institute on Aging, National Institutes of Health, Bethesda, Maryland, 20892, United States of America; University of Nebraska Medical Center, UNITED STATES

## Abstract

Metabolic homeostasis is critical for all biological processes in the brain. The metabolites are considered the best indicators of cell states and their rapid fluxes are extremely sensitive to cellular changes. While there are a few studies on the metabolomics of Parkinson’s disease, it lacks longitudinal studies of the brain metabolic pathways affected by aging and the disease. Using ultra-high performance liquid chromatography and tandem mass spectroscopy (UPLC/MS), we generated the metabolomics profiling data from the brains of young and aged male PD-related **α**-synuclein A53T transgenic mice as well as the age- and gender-matched non-transgenic (nTg) controls. Principal component and unsupervised hierarchical clustering analyses identified distinctive metabolites influenced by aging and the A53T mutation. The following metabolite set enrichment classification revealed the alanine metabolism, redox and acetyl-CoA biosynthesis pathways were substantially disturbed in the aged mouse brains regardless of the genotypes, suggesting that aging plays a more prominent role in the alterations of brain metabolism. Further examination showed that the interaction effect of aging and genotype only disturbed the guanosine levels. The young A53T mice exhibited lower levels of guanosine compared to the age-matched nTg controls. The guanosine levels remained constant between the young and aged nTg mice, whereas the aged A53T mice showed substantially increased guanosine levels compared to the young mutant ones. In light of the neuroprotective function of guanosine, our findings suggest that the increase of guanosine metabolism in aged A53T mice likely represents a protective mechanism against neurodegeneration, while monitoring guanosine levels could be applicable to the early diagnosis of the disease.

## Introduction

Parkinson’s disease (PD) is the most common degenerative movement disorder [1]. Although the metabolites are very sensitive to cellular changes and serve as the best indicators of cell states [2], only a few studies have used metabolomics to evaluate metabolic signatures and pathways involved in PD. The pentose phosphate pathway has been reported as being affected the most in the postmortem brains of PD patients [3]. While the urate levels are significantly reduced, the glutathione is significantly increased in the plasma of PD patients [4–6]. However, there lack longitudinal studies for monitoring the alterations of metabolites in the brain during the progression of the disease. In addition, the impact of aging itself on the brain metabolism remains to be determined.

The A53T missense mutation of **α**-synuclein is the first genetic causal factor linked to PD [7]. In a line of **α**-synuclein transgenic mice [8], overexpression of human **α**-synuclein A53T mutation recapitulates two canonical PD-related neuropathological abnormalities, namely progressive loss of nigrostriatal dopaminergic neurons and formation **α**-synuclein-positive aggregates [9]. In addition to modeling PD-related neuron loss, these A53T transgenic mice could be also useful in studying the metabolic pathways affected by PD and aging.

To understand how the PD-related **α**-synuclein A53T mutation alters brain metabolism during aging, the brains of 3-month (young) and 18-month-old (aged) A53T transgenic mice and age-matched non-transgenic (nTg) controls were collected for metabolomics profiling. A series of statistical analyses of the profiling data revealed multiple metabolic pathways affected by aging and the disease.

## Materials and Methods

### Ethics statement

All mouse work follows the guidelines approved by the Institutional Animal Care and Use Committees of the National Institute of Child Health and Human Development, US National Institutes of Health.

### Transgenic mice

The *Pitx3-IRES2-tTA*/tetO-A53T bigenic mice were generated as previously described [8]. Mice were housed in a 12h light/dark cycle and fed regular diet libitum. Four groups of mice were used for this study: 3-month and 18-month-old nTg mice, and 3-month and 18-month-old A53T bigenic mice. Each group had eight male mice. The forebrain and midbrain tissues were collected for global untargeted metabolic profiling by Metabolon Inc. (Durham, NC).

### Sample preparation

Samples were prepared using the automated MicroLab STAR system from Hamilton Company. A recovery standard was added prior to the first step in the extraction process for QC purposes. Sample preparation was conducted using aqueous methanol extraction process to remove the protein fraction while allowing maximum recovery of small molecules. The resulting extract was divided into four fractions: one for analysis by UPLC/MS/MS (positive mode), one for UPLC/MS/MS (negative mode), one for GC/MS, and one for backup. Samples were placed briefly on a TurboVap (Zymark) to remove the organic solvent. Each sample was then frozen and dried under vacuum. Samples were then prepared for the appropriate instrument, either UPLC/MS/MS or GC/MS.

### Ultrahigh performance liquid chromatography/tandem Mass Spectroscopy

The UPLC/MS/MS portion of the platform was based on a Waters ACQUITY ultra-performance liquid chromatography (UPLC) and a Thermo-Finnigan linear trap quadrupole (LTQ) mass spectrometer, which consisted of an electrospray ionization (ESI) source and linear ion-trap (LIT) mass analyzer. The sample extract was dried then reconstituted in acidic or basic LC-compatible solvents, each of which contained 8 or more injection standards at fixed concentrations to ensure injection and chromatographic consistency. One aliquot was analyzed using acidic positive ion optimized conditions and the other using basic negative ion optimized conditions in two independent injections using separate dedicated columns. Extracts reconstituted in acidic conditions were gradient eluted using water and methanol containing 0.1% formic acid, while the basic extracts, which also used water/methanol, contained 6.5mM Ammonium Bicarbonate. The MS analysis alternated between MS and data-dependent MS2 scans using dynamic exclusion. Raw data files are archived and extracted as described below.

### Gas chromatography/Mass Spectroscopy

The samples destined for GC/MS analysis were re-dried under vacuum desiccation for a minimum of 24h prior to being derivatized under dried nitrogen using bistrimethyl-silyl-triflouroacetamide (BSTFA). The GC column was 5% phenyl and the temperature ramp was from 40° to 300°C in a 16 minute period. Samples were analyzed on a Thermo-Finnigan Trace DSQ fast-scanning single-quadrupole mass spectrometer using electron impact ionization. The instrument was tuned and calibrated for mass resolution and mass accuracy on a daily basis. Raw data files are archived and extracted as described below.

### Data extraction and compound identification

Raw data was extracted, peak-identified and QC processed using Metabolon’s hardware and software. These systems are built on a web-service platform utilizing Microsoft’s.NET technologies, which run on high-performance application servers and fiber-channel storage arrays in clusters to provide active failover and load-balancing. Compounds were identified by comparison to library entries of purified standards or recurrent unknown entities. More than 3500 commercially available purified standard compounds have been acquired and registered into LIMS for distribution to both the LC and GC platforms for determination of their analytical characteristics.

### Statistics and bioinformatics

Missing values (if any) are assumed to be below the level of detection. However, metabolites that were detected in all samples from one or more groups, but not in samples from other groups were assumed to be near the lower limit of detection in the groups in which they were not detected. In this case, the lowest detected level of these metabolites was imputed for samples in which that biochemical was not detected. Following scale normalization and imputation with minimum observed values for each compound, two-way ANOVA and one-way ANOVA tests were used to identify metabolites that differed significantly between experimental groups. Principal component analysis was performed using R, a statistical computing environment (www.r-project.org). Unsupervised hierarchical clustering was performed using complete linkage and Pearson rank correlation distance on the normalized metabolites in R. The web-based metabolomics data processing tool MetaboAnalyst 2.0 was used for pathway and metabolite set enrichment analysis (MSEA) analysis. See http://www.metaboanalyst.ca for detailed methodology [10]. The database MetaboAnalyst 2.0 used for the metabolic pathway analysis is Human Metabolom database (HMDB), Small Molecule Pathway Database (SMPDB) and Kyoto Encyclopedia of Genes and Genomes (KEGG) [10]. Beside two-way ANOVA, Random forest (RF) classification method in R package was applied to the scaled and imputed metabolite data; the RF method was implemented with Breiman’s random forest algorithm [11]. RF is a machine learning method. Comparing to other methods applied in analyzing metabolomics data, such as projection to latent structures (PLS), support vector machine (SVM) and linear discriminant analysis (LDA), RF has simple theory, fast speed and is insensitive to noise. RF is getting more frequent in metabolomics data analysis [12]. Values of mean decrease in accuracy calculated from RF classification analysis were used to identify the metabolites that are significantly affected by aging. Mean Decrease Accuracy is a score used to describe how importantly a compound contributes to the group classification. It measures how much classification error would be reduced when the certain metabolite was counted in the model. Therefore the metabolites with a higher mean decrease in accuracy are more important for the classification of the data [11].

## Results

### Aging exerts more significant impact on the alterations of metabolites

**Fig 1:**
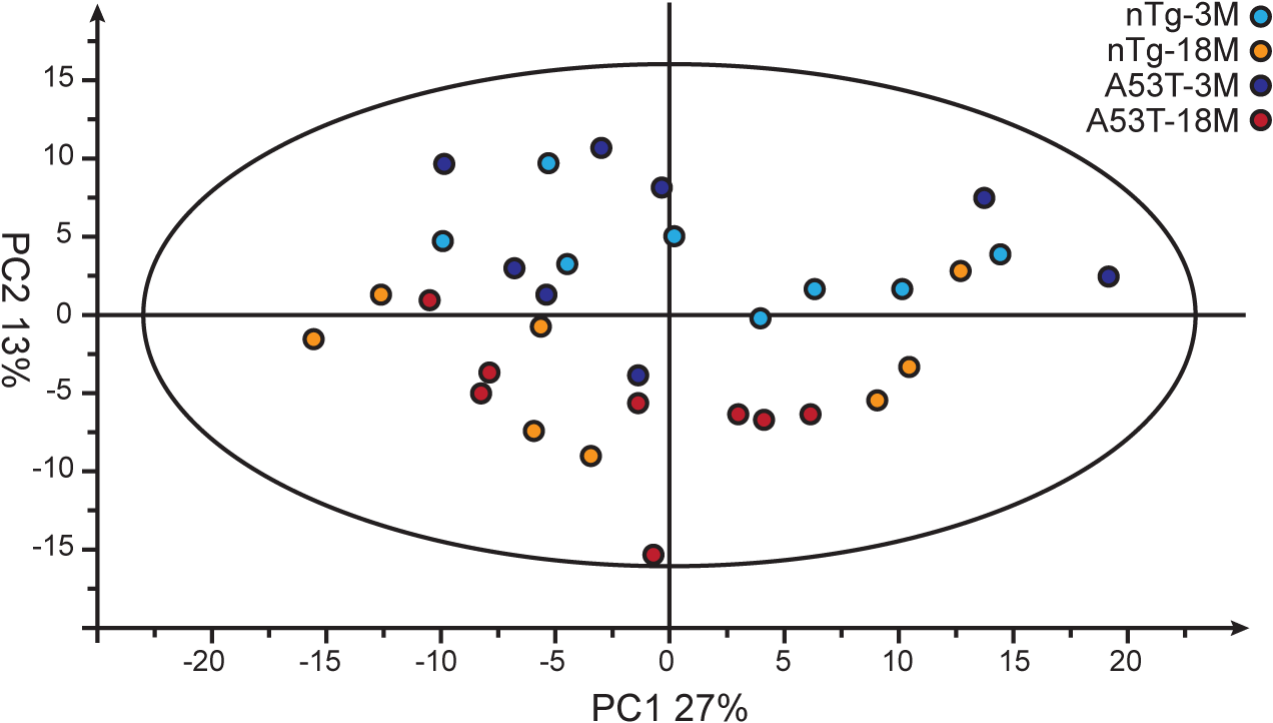
Principle component analysis (PCA) of brain metabolites influenced by aging and Parkinson’s disease-related α-synuclein A53T mutation. PCA plot showing a segregation of the metabolites affected in all the aged mouse brain samples (colored by dark and light red for A53T and nTg mice) from the young ones (colored by dark and light blue for A53T and nTg mice). The first principle component (PC1) accounts for 27% of the overall variability; the second principle component (PC2) accounts for 13% of the overall variability.

The principal component analysis (PCA) was used to quantitatively compare the global metabolomics profiling data collected from the four groups of mice. Based on the top two principal components, the aged mice appeared as a separate group from the young ones, whereas the A53T bigenic groups could not be distinguished from the nTg groups (Fig 1).

**Fig 2:**
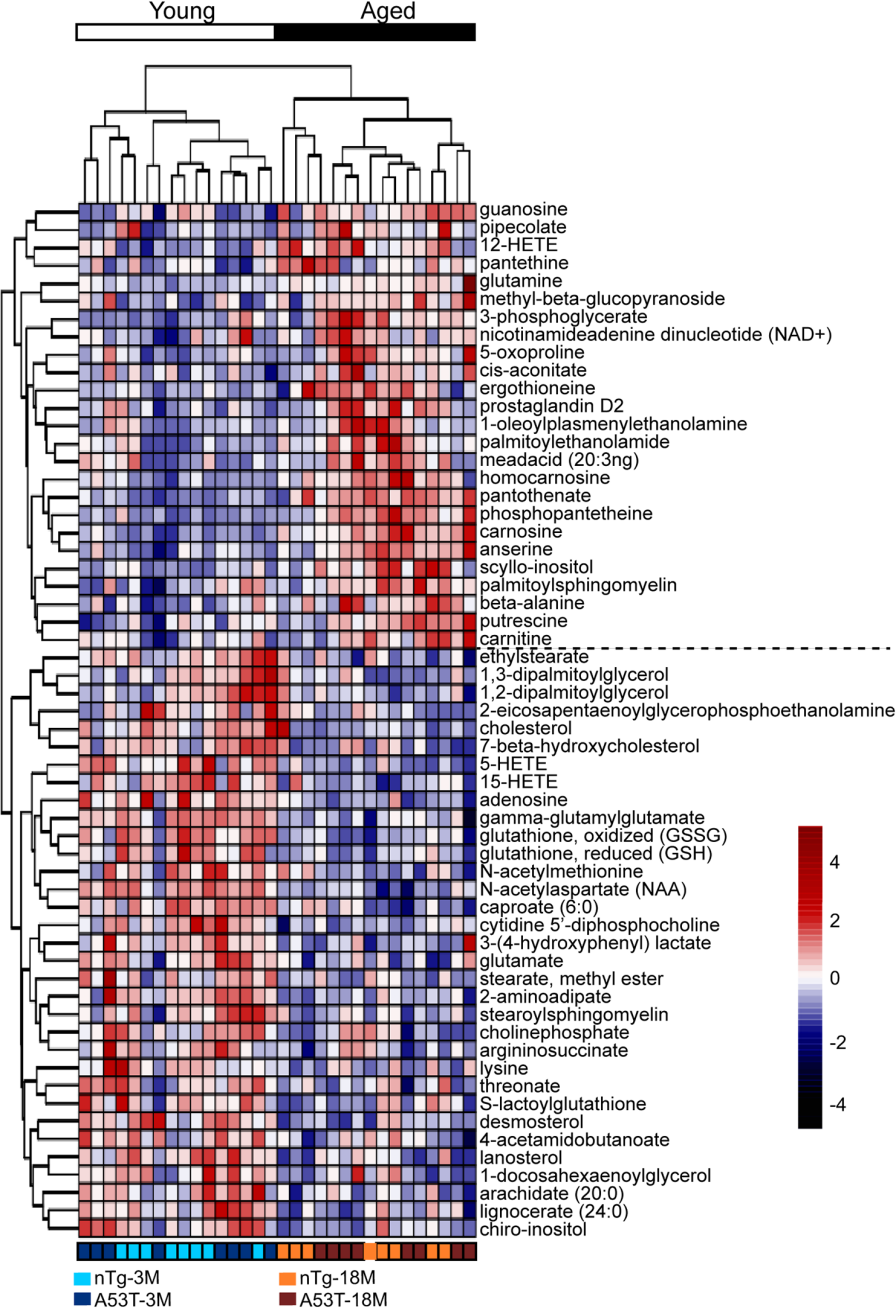
Hierarchical clustering of metabolites affected by aging. There are 58 metabolites significantly affected by aging from a two-way ANOVA test. The unsupervised hierarchical clustering plot shows that an age-dependent segregation of these metabolites. The scaled intensity of 58 metabolites is relatively depicted according to the color key shown on the right. Red indicates high intensity levels; blue, low intensity levels.

We subsequently identified the metabolites with significant changes between young and aged mice using a false discovery rate (FDR) of q < 0.05 in two-way ANOVA test, a commonly used filtering criterion for large-scale data analysis [13]. The test allowed us to capture the maximum number of compounds that potentially changed between groups. We observed that 58 metabolites were significantly disturbed during aging (Fig 2). Among them, 25 metabolites (8.7%) were increased, whereas 33 metabolites (11.4%) were decreased in the aged mice. Using hierarchical clustering on the profile of these 58 metabolites, the mice were strictly separated into large groups according to age difference and several interesting metabolite clusters became apparent as well (Fig 2). One of these clusters contained alanine and its products. The alanine metabolism pathway reflected energy demand in mouse brain [14]. In addition, various intermediates and products of oxidation-reduction pathway were altered, such as oxidized and reduced glutathione that both decreased in the aged brains (Fig 2).

**Fig 3:**
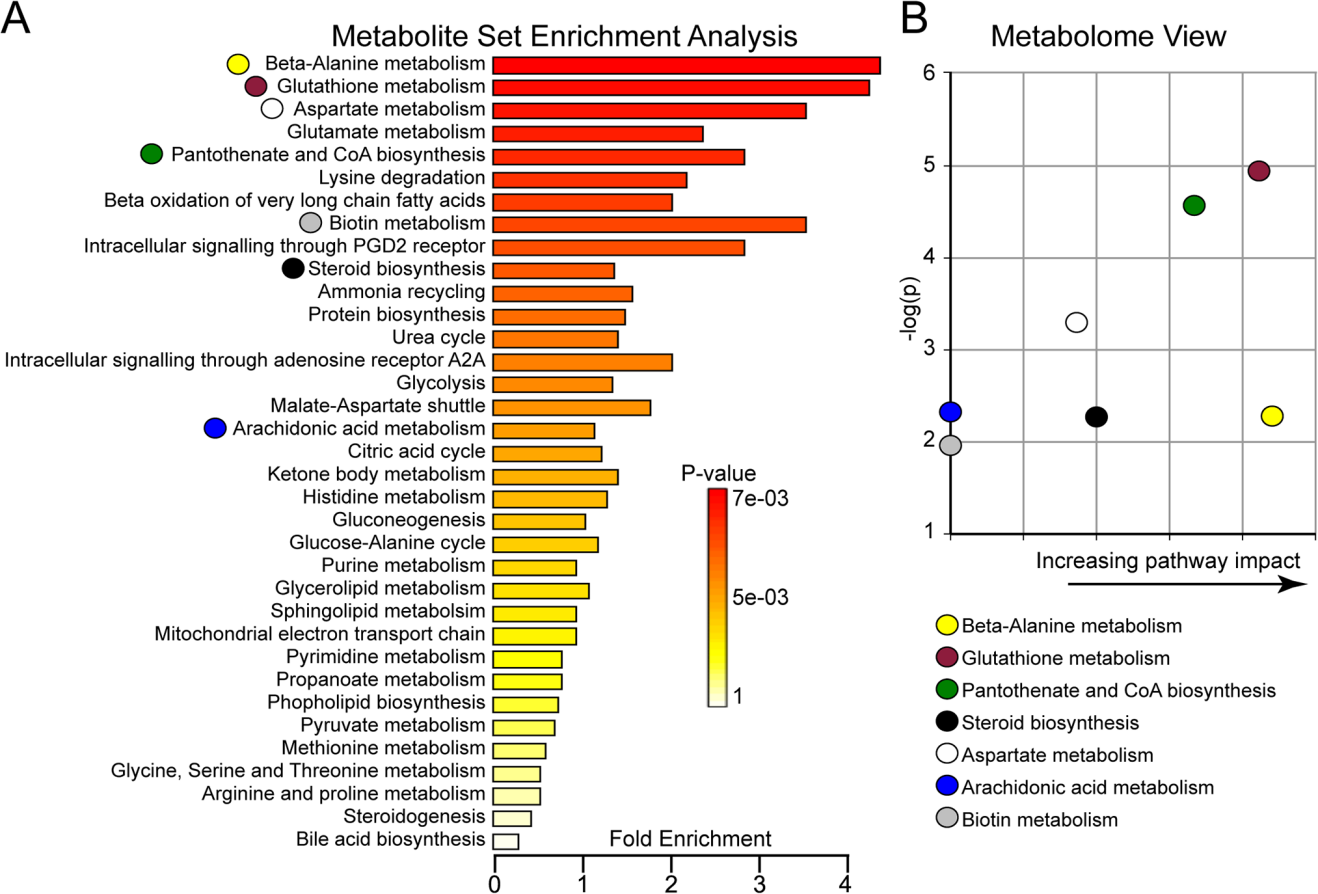
Metabolite pathway affected by aging. (A) Summary plot for the metabolite set enrichment analysis (MSEA) are ranked by Holm p-value. Holm p-value is the p value adjusted by Holm-Bonferroni test that is a method to counteract the problem of multiple comparisons and is widely used for large-scale data analysis. (B) Metabolome view shows key nodes in metabolic pathways that have been significantly altered with aging. The y-axis represents unadjusted p value from pathway enrichment analysis. The x-axis represents increasing metabolic pathway impact according to the betweenness centrality from pathway topology analysis.

Following hierarchical cluster analysis, we performed metabolite set enrichment analysis (MSEA) to identify which pathways were affected by those 58 distinguishing metabolites. Our results indicated that the best metabolites to predict age affect were in redox homeostasis, lipid synthesis and amino acid metabolism pathways (Fig 3A). In addition, we performed the metabolome view analysis to demonstrate whether a metabolite node was affected. We confirmed the finding that the β-alanine metabolism, glutathione metabolism and pantothenate and CoA biosynthesis pathways were disturbed in the aged mouse brains (Fig 3B).

### Identification of signature metabolites as biomarkers of aged brains

**Fig 4:**
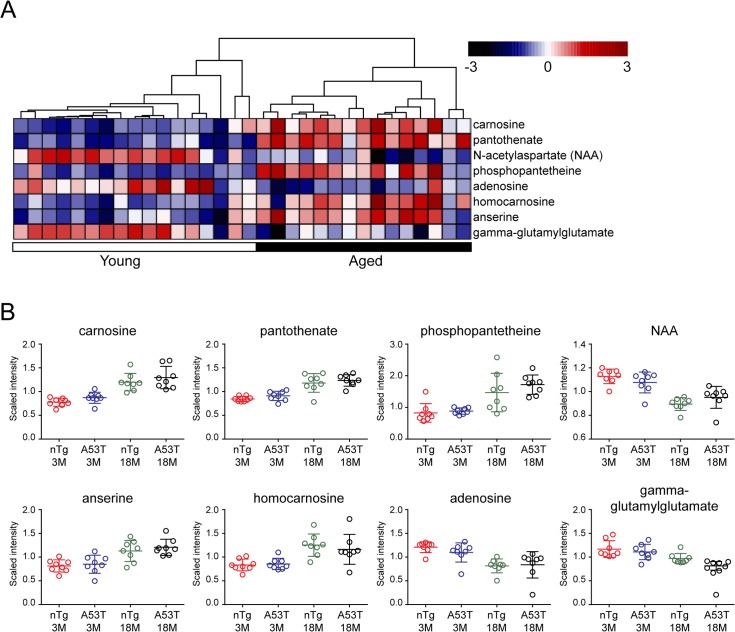
Identification of Aging-related metabolite biomarker. (A) Two-way ANOVA test (q value <0.05) and RF analysis (Mean decrease accuracy >5) identify eight metabolites significantly affected by aging. Unsupervised hierarchical clustering plot shows the segregation between aged and young samples. The scaled intensity of eight metabolites is relatively depicted according to the color key shown on the top. Red indicates high intensity levels; blue, low intensity levels. The q-value used here represents the measurement of the proportion of false positives incurred (also called the false discovery rate). (B) Scatter plots compare the scaled intensity of those eight metabolites from different sample groups.

The metabolic differences between young and aged mice led us to test whether they can be used as biomarkers to reliably and accurately classify the two age groups. To separate different groups of samples by selection of a subset of signature metabolites or genes is applied widely with large-scale data sets, such as metabolomics and gene expression analyses [15, 16]. Accordingly, we first conducted one-way ANOVA test separately with nTg and A53T two genetic subgroups. In the nTg subgroup, there were 10 metabolites significantly changed in the aged mice comparing to the young ones, while in the A53T subgroup 24 metabolites were differentially altered in the aged animals (Table 1 and S1 Data). After intersecting the set of 10 metabolites from nTg and 24 metabolites from A53T, we found four overlapping metabolites: carnosine, N-acetylaspartate (NAA), pantothenate and phosphopantetheine (Table 1). We also did the Random Forests (RF) analysis to validate the results from one-way ANOVA test. RF is one such learning algorithm that can discriminate the two groups by estimating the importance of each variable to the classification [11]. The “mean decrease accuracy” indicated how much a certain metabolite contributes to separation of the two test groups [11]. We conducted RF analysis with the same two genetic subgroups nTg and A53T as one-way ANOVA test. In the nTg subgroup, there were 19 metabolites significantly changed in the aged mice compared to the young ones, while for A53T mice, totally 23 metabolites were differentially altered in the aged animals (Table 2 and S1 Data). We did the intersection as well with the sets of 19 metabolites and 23 metabolites. Finally eight overlapping metabolites were identified (Fig 4A). Among them, four metabolites were identified by the ANOVA test as shown above, and the other four were homocarnosine, anserine, gamma-glutamylglutamate and adenosine. Following one-way ANOVA test and RF analysis, we performed hierarchical clustering on these eight metabolites. As expected, all mouse samples can be separated reliably based on the age (Fig 4A). Therefore, these metabolites show strong predictive value on the age factors regardless of genotype difference.

**Table 1:** A list of metabolites substantially affected by aging based on the one-way ANOVA test.

	Metabolite Name	q-value	Super-pathway
nTg (18M vs. 3M)	Carnosine	0.0004	Amino acid
	N-acetylaspartate (NAA)	0.0004	Amino acid
	Pantothenate	0.0013	Cofactor
	3-(4-hydroxyphenyl) lactate	0.0163	Amino acid
	Homocarnosine	0.0163	Amino acid
	Phosphopantetheine	0.0176	Cofactor
	glutathione, reduced	0.0177	Amino acid
	Fructose	0.0276	Carbohydrate
	cytidine 5’-diphophocholine	0.0401	Lipid
	scyllo-inositol	0.0485	Lipid
A53T (18M vs. 3M)	Guanosine	0.0005	Nucleotide
	Carnosine	0.0012	Amino acid
	Pantothenate	0.0023	Cofactor
	Phosphopantetheine	0.0026	Cofactor
	Adenosine	0.0030	Nucleotide
	Desmosterol	0.0038	Lipid
	lignocerate (24:0)	0.0046	Lipid
	5-HETE	0.0046	Lipid
	stearate, methyl ester	0.0065	Lipid
	Putrescine	0.0111	Amino acid
	Anserine	0.0155	Amino acid
	3-phosphoglycerate	0.0175	Carbohydrate
	Cholesterol	0.0209	Lipid
	dehydroascorbate	0.0209	Cofactor
	chiro-inositol	0.0215	Lipid
	threonate	0.0215	Cofactor
	glucose-6-phosphate	0.0351	Carbohydrate
	caproate (6:0)	0.0363	Lipid
	ethyl stearate	0.0388	Lipid
	1-myristoylglycerophosphocholine	0.0388	Lipid
	2-oleoylglycerophosphoserine	0.0388	Lipid
	N-acetylaspartate (NAA)	0.0413	Amino acid
	mannose-6-phosphate	0.0461	Carbohydrate
	2-aminoadipate	0.0470	Amino acid

**Table 2:** A list of metabolites substantially affected by aging based on the Random Forests test.

	Metabolite name	Mean decrease accuracy	Super-pathway
nTg (18M vs. 3M)	glutathione, oxidized	16.27	Amino acid
	N-acetylaspartate (NAA)	16.08	Amino acid
	carnosine	15.76	Amino acid
	glutathione, reduced	15.26	Amino acid
	gamma-glutamylglutamate	12.77	Amino acid
	glutamine	12.49	Amino acid
	3-(4-hydroxyphenyl) lactate	12.35	Amino acid
	pantothenate	11.18	Cofactor
	cytidine 5’-diphosphocholine	10.38	Lipid
	adenosine 5’-monophosphate	9.48	Nucleotide
	lanosterol	9.09	Lipid
	homocarnosine	8.89	Amino acid
	lysine	7.18	Amino acid
	2-aminoadipate	6.71	Amino acid
	phosphopantetheine	6.34	Cofactor
	12-HETE	6.03	Lipid
	anserine	5.80	Amino acid
	leucine	5.46	Amino acid
	adenosine	5.18	Nucleotide
A53T (18M vs. 3M)	guanosine	15.76	Nucleotide
	phosphopantetheine	15.60	Cofactor
	desmosterol	15.38	Lipid
	pipecolate	14.19	Amino acid
	pantothenate	11.79	Cofactor
	carnosine	11.40	Amino acid
	sucrose	9.70	Carbohydrate
	scyllo-inositol	9.65	Lipid
	5-HETE	9.61	Lipid
	adenosine	9.60	Nucleotide
	ergothioneine	9.59	Xenobiotics
	stearate, methyl ester	9.33	Lipid
	dehydroascorbate	9.17	Cofactor
	lignocerate (24:0)	9.08	Lipid
	anserine	8.97	Amino acid
	cholesterol	8.92	Lipid
	putrescine	5.85	Amino acid
	homocarnosine	5.68	Amino acid
	Palmitoyl ethanolamide	5.67	Lipid
	chiro-inositol	5.63	Lipid
	N-acetylaspartate (NAA)	5.61	Amino acid
	3-phosphoglycerate	5.03	Carbohydreate
	gamma-glutamylglutamate	5.00	Amino acid

Carnosine is a dipeptide, composed of β-alanine and ι-histidine, an important compound involved in alanine metabolism [17]. In mammals, carnosine has high concentration in the skeletal muscle and the olfactory bulb [18]. Comparing to the extensive studies on muscle tissues, the functions of carnosine in brain are poorly understood [17]. Carnosine has been proposed to protect brains mainly through antioxidant, metal chelating, and antiglycative properties, like what it does in the muscles [19]. Homocarnosine, a carnosine analog, is more prevalent than carnosine in the mammalian brain [17]. Anserine is the most common variant of methylated carnosine analogs [17]. Our data showed that carnosine, homocarnosine and anserine were all highly accumulated in the aged mouse brains (Fig 4B), which may be related with their antioxidant activities to protect brain from excessive ROS stress during aging.

NAA is localized primarily in neurons and is considered to be a putative neuronal marker [20]. Consistent with the previous studies [21, 22], we observed reduced NAA in the aged mice (Fig 4B). In addition, some recent research suggests that there is a strong positive correlation between concentrations of NAA and glutamate [23]. In our study, the levels of glutamate were also decreased in the aged mice like NAA (Fig 2). In contrast to glutamate reduction, glutamine concentration was somewhat increased in the aged mice. Glutamine is localized primarily in astrocytes, where glutamine synthetase is considered to be a marker of glial activity [24]. Therefore, the finding of increased glutamine levels could be indicative of glial proliferation (Fig 2), which often accompanies with neurodegeneration [23].

### Metabolite guanosine shows age-dependent alterations in A53T bigenic mice

**Fig 5:**
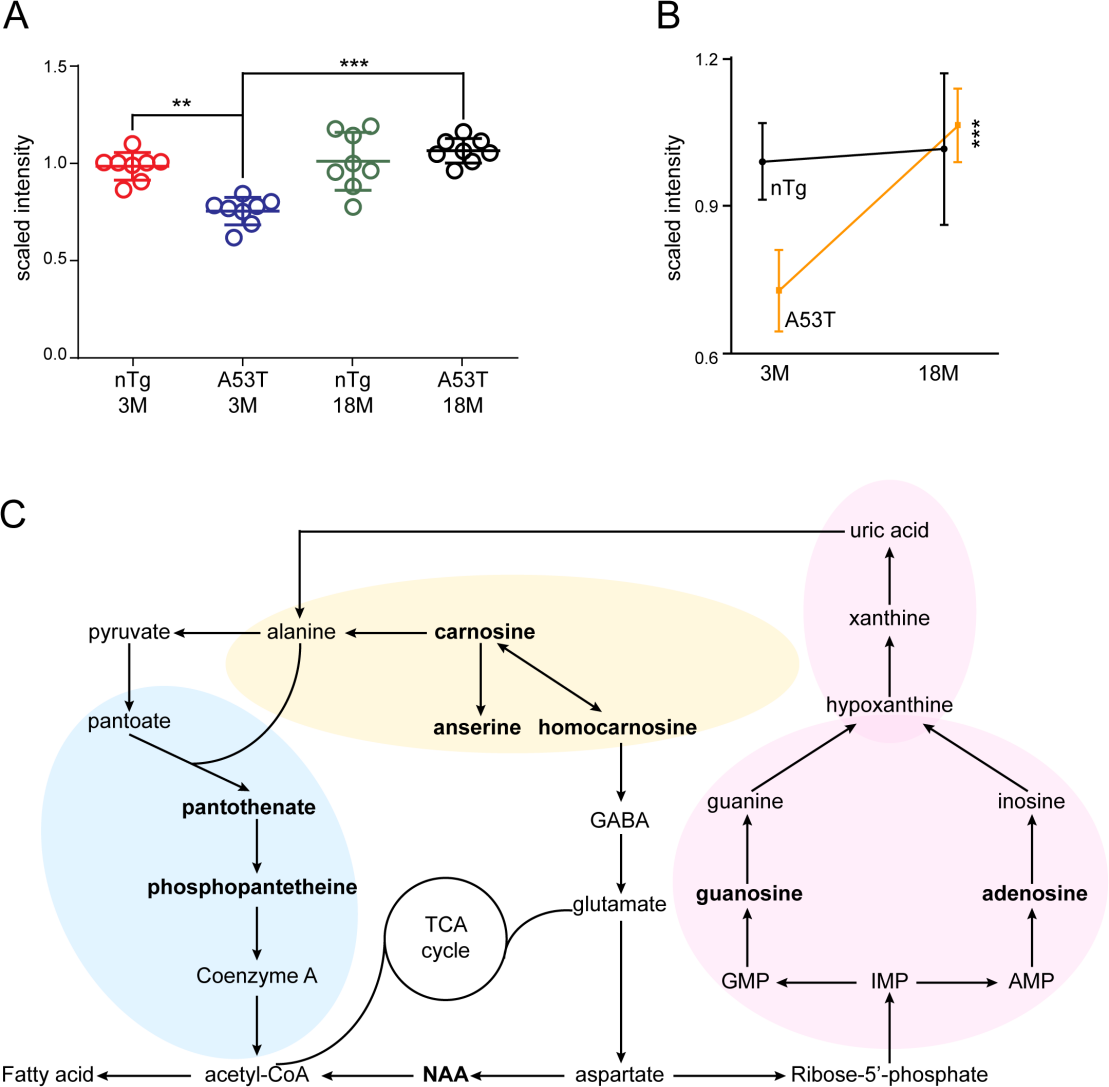
Guanosine metabolism is affected by both aging and A53T mutation. (A) Scatter plot depicts the alteration of guanosine levels in different age and genotype groups. One-way ANOVA test, **q value <0.01; ***q value <0.001. (B) Line graph highlights the age-dependent changes of guanosine in A53T and nTg mice. One-way ANOVA test, ***q value <0.001. (C) Schematic diagram summarizes the alanine metabolic (in yellow shade) and acetyl-CoA biosynthesis (in blue shade) pathways mainly affected by aging, and the purine metabolic (in pink shade) pathway influenced by both aging and genotypes. The metabolites highlighted with the bold font represent the ones differentially altered between groups (q value < 0.05 in two-way ANOVA test).

In the two-way ANOVA test, no metabolite showed significant alteration between A53T bigenic and nTg mice depending on genotype effects (S1 Data). We then conducted one-way ANOVA test with two age subgroups separately to identify the impact of genotypes on metabolites. In the young subgroup, only one metabolite guanosine was significantly altered in the A53T mice compared to the nTg ones (Fig 5A and 5B). In the aged subgroup, however, no metabolite showed differential alterations between two genotypes (S1 Data). Further data analyses revealed that guanosine accumulated more in the aged A53T mice compared to the young ones, whereas, no substantial alterations of guanosine levels were found in the young and aged nTg subgroups (Fig 5A and 5B). Therefore, guanosine was affected by a combination of genotype and age factors, as confirmed by the two-way ANOVA interaction test (S1 Data).

## Discussion

Excessive reactive oxygen species (ROS) generated by oxidative reactions within the mitochondria has been implicated in many age-related neurological diseases, such as PD, Alzheimer’s disease, and amyotrophic lateral sclerosis (ALS) [25, 26]. Reduced glutathione (GSH) is able to scavenge both ROS and xenobiotics within the cells, which leads to form the oxidized glutathione (GSSG), GSSG can then be returned to GSH by glutathione reductase (GSR) [27]. Here we found that both GSH and GSSG levels were decreased significantly in the aged mice (Fig 2). Decreased GSH in aged mice has been reported previously [28, 29]. A high GSH/GSSG ratio represents a properly functioning glutathione system observed normally in young animals, while a low GSH/GSSG ratio associates with aging models [30, 31]. In our study, the ratio of GSH to GSSG was not altered by either age or genotype (S1 Fig). The ratios of GSH to GSSG in four subgroups were all close to one. By contrast, previous studies show the levels of GSH are much higher than GSSG in the both young and old mouse brains [28, 32]. We speculate that the LC/MS platform used in our study cannot accurately detect GSH or some GSH was oxidized into GSSG during the preparation and storage of samples.

β-alanine is the β form of the amino acid alanine, which is intimately linked with glutamate/glutamine cycling [33]. The ammonia from glutamate/glutamine transition in neurons is carried by alanine and leucine and returned to the astrocyte [34]. After deamination, alanine in the astrocyte is largely converted into lactate, which is released into the extracellular space and taken up by neurons [35, 36]. Many studies indicate that lactate is a main energy source for neurons and its utilization is necessary for long-term memory formation [36, 37]. Our results showed increased alanine levels in the aged mouse brains compared to the young ones, suggesting a possible compensatory mechanism in the aged brains to make up for the less energy production.

Two reasons may be considered for the lack of metabolites affected by genotypes according to two-way ANOVA analysis. One is the large variation of metabolite data sets from different subgroups. The other is that the metabolic changes mainly occur in a small fraction of neurons, such as the nigrostriatal dopaminergic neurons, which is likely undetectable when the whole brain is used for the assay. The in situ studies of brain metabolomics using Magnetic Resonance Spectroscopy (MRS) and positron emission tomography could be useful in identifying metabolite alterations in small brain regions.

Guanosine is a guanine-based purine and can be phosphorylated to become several different forms (GMP, GDP and GTP), which affect multiple cellular processes, including cellular growth, differentiation and survival [38, 39]. Guanosine can exert protective effects against staurosporine- or β-amyloid-induced apoptosis [40, 41]. Recently the neuroprotective effects of guanosine in the central nervous system have also been identified in a PD-related cellular model system, which shows guanosine effectively prevent 1-methyl-4-phenylpyridinium (MPP^+^)-induced PC12 cell apoptosis by stabilizing the mitochondrial membrane potential [42]. MPP^+^ induction can cause DA neurons damage and result in pathological symptoms similar to PD [43]. Guanosine also protects 6-hydroxydopamine (6-OHDA) treated SH-SY5Y cells (6-OHDA is widely used to mimic neuropathology of PD) to promote their survival through the apoptotic signaling pathway, including p-38, c-Jun N-terminal kinase (JNK) and protein kinase B [41]. In the A53T mice, the DA neurodegeneration starts as early as 1 month of age [8], which is correlated with a significant reduction of guanosine levels in the young A53T mice, suggesting that without the sufficient guanosine protection, the DA neurons are likely more vulnerable to the mutant **α**-synuclein-induced cytotoxicity. However, the underlying mechanism for the neuroprotective function of guanosine in A53T bigenic mice remains to be clarified.

## Conclusion

In the present study we identified eight aging-related metabolite biomarkers, which are mainly involved in the alanine metabolism and acetyl-CoA biosynthesis pathways (Fig 5C). These two pathways interconnect closely and both contribute to the cellular energy homeostatic [14, 44]. Furthermore, our results demonstrate that purine metabolic pathway was disturbed by a combination of the age and genotype factors (Fig 5C). Urate, the end product of this pathway, has been considered as a biomarker for PD diagnosis [45]. We therefore suggest the purine pathway likely contribute to neuroprotection against DA neuron loss in PD.

## Supporting Information

S1 Data The data sheet contains the scaled raw metabolic data. (XLSX)

S1 Fig The ratios of GSH to GSSG are presented in four different subgroups. (TIF)

## References

[1] Thomas B, Beal MF. Parkinson's disease. Hum Mol Genet. 2007;16 Spec No. 2:R183–94. Epub 2007/10/04. 10.1093/hmg/ddm159 . 17911161

[2] Cascante M, Llorens M, Melendez-Hevia E, Puigjaner J, Montero F, Marti E. The metabolic productivity of the cell factory. Journal of theoretical biology. 1996;182(3):317–25. . 8944164 10.1006/jtbi.1996.0170

[3] Poliquin PO, Chen J, Cloutier M, Trudeau LE, Jolicoeur M. Metabolomics and in-silico analysis reveal critical energy deregulations in animal models of Parkinson's disease. PLoS One. 2013;8(7):e69146 10.1371/journal.pone.0069146 23935941 PMC3720533

[4] O'Reilly EJ, Gao X, Weisskopf MG, Chen H, Schwarzschild MA, Spiegelman D, et al Plasma urate and Parkinson's disease in women. American journal of epidemiology. 2010;172(6):666–70. 10.1093/aje/kwq195 20682521 PMC2950819

[5] Schwarzschild MA, Schwid SR, Marek K, Watts A, Lang AE, Oakes D, et al Serum urate as a predictor of clinical and radiographic progression in Parkinson disease. Archives of neurology. 2008;65(6):716–23. 10.1001/archneur.2008.65.6.nct70003 18413464 PMC2574855

[6] Ascherio A, LeWitt PA, Xu K, Eberly S, Watts A, Matson WR, et al Urate as a predictor of the rate of clinical decline in Parkinson disease. Archives of neurology. 2009;66(12):1460–8. 10.1001/archneurol.2009.247 19822770 PMC2795011

[7] Polymeropoulos MH, Lavedan C, Leroy E, Ide SE, Dehejia A, Dutra A, et al Mutation in the alpha-synuclein gene identified in families with Parkinson's disease. Science. 1997;276(5321):2045–7. Epub 1997/06/27. . 9197268 10.1126/science.276.5321.2045

[8] Lin X, Parisiadou L, Sgobio C, Liu G, Yu J, Sun L, et al Conditional expression of Parkinson's disease-related mutant alpha-synuclein in the midbrain dopaminergic neurons causes progressive neurodegeneration and degradation of transcription factor nuclear receptor related 1. J Neurosci. 2012;32(27):9248–64. Epub 2012/07/06. 10.1523/JNEUROSCI.1731-12.2012 22764233 PMC3417246

[9] Spillantini MG, Schmidt ML, Lee VM, Trojanowski JQ, Jakes R, Goedert M. Alpha-synuclein in Lewy bodies. Nature. 1997;388(6645):839–40. Epub 1997/08/28. 10.1038/42166 . 9278044

[10] Xia J, Mandal R, Sinelnikov IV, Broadhurst D, Wishart DS. MetaboAnalyst 2.0—a comprehensive server for metabolomic data analysis. Nucleic acids research. 2012;40(Web Server issue):W127–33. 10.1093/nar/gks374 22553367 PMC3394314

[11] Breiman L. Random forests. Mach Learn. 2001;45(1):5–32. 10.1023/A:1010933404324 .

[12] Touw WG, Bayjanov JR, Overmars L, Backus L, Boekhorst J, Wels M, et al Data mining in the Life Sciences with Random Forest: a walk in the park or lost in the jungle? Briefings in bioinformatics. 2013;14(3):315–26. 10.1093/bib/bbs034 22786785 PMC3659301

[13] Rhodes DR, Yu J, Shanker K, Deshpande N, Varambally R, Ghosh D, et al Large-scale meta-analysis of cancer microarray data identifies common transcriptional profiles of neoplastic transformation and progression. Proceedings of the National Academy of Sciences of the United States of America. 2004;101(25):9309–14. 10.1073/pnas.0401994101 15184677 PMC438973

[14] Broer S, Broer A, Hansen JT, Bubb WA, Balcar VJ, Nasrallah FA, et al Alanine metabolism, transport, and cycling in the brain. J Neurochem. 2007;102(6):1758–70. 10.1111/j.1471-4159.2007.04654.x . 17504263

[15] LeWitt P. Recent advances in CSF biomarkers for Parkinson's disease. Parkinsonism Relat Disord. 2012;18 Suppl 1:S49–51. 10.1016/S1353-8020(11)70017-7 . 22166453

[16] Pal NR, Aguan K, Sharma A, Amari S. Discovering biomarkers from gene expression data for predicting cancer subgroups using neural networks and relational fuzzy clustering. BMC bioinformatics. 2007;8:5 10.1186/1471-2105-8-5 17207284 PMC1770936

[17] Boldyrev AA, Aldini G, Derave W. Physiology and pathophysiology of carnosine. Physiological reviews. 2013;93(4):1803–45. 10.1152/physrev.00039.2012 . 24137022

[18] Biffo S, Grillo M, Margolis FL. Cellular localization of carnosine-like and anserine-like immunoreactivities in rodent and avian central nervous system. Neuroscience. 1990;35(3):637–51. . 2199844 10.1016/0306-4522(90)90335-2

[19] Bonfanti L, Peretto P, De Marchis S, Fasolo A. Carnosine-related dipeptides in the mammalian brain. Prog Neurobiol. 1999;59(4):333–53. . 10501633 10.1016/s0301-0082(99)00010-6

[20] Nie K, Zhang Y, Huang B, Wang L, Zhao J, Huang Z, et al Marked N-acetylaspartate and choline metabolite changes in Parkinson's disease patients with mild cognitive impairment. Parkinsonism Relat Disord. 2013;19(3):329–34. 10.1016/j.parkreldis.2012.11.012 . 23238068

[21] Brooks JC, Roberts N, Kemp GJ, Gosney MA, Lye M, Whitehouse GH. A proton magnetic resonance spectroscopy study of age-related changes in frontal lobe metabolite concentrations. Cereb Cortex. 2001;11(7):598–605. . 11415962 10.1093/cercor/11.7.598

[22] Schuff N, Ezekiel F, Gamst AC, Amend DL, Capizzano AA, Maudsley AA, et al Region and tissue differences of metabolites in normally aged brain using multislice 1H magnetic resonance spectroscopic imaging. Magnetic resonance in medicine: official journal of the Society of Magnetic Resonance in Medicine / Society of Magnetic Resonance in Medicine. 2001;45(5):899–907. 11323817 10.1002/mrm.1119 PMC1851682

[23] Kaiser LG, Schuff N, Cashdollar N, Weiner MW. Age-related glutamate and glutamine concentration changes in normal human brain: 1H MR spectroscopy study at 4 T. Neurobiology of aging. 2005;26(5):665–72. 10.1016/j.neurobiolaging.2004.07.001 15708441 PMC2443746

[24] Martinez-Hernandez A, Bell KP, Norenberg MD. Glutamine synthetase: glial localization in brain. Science. 1977;195(4284):1356–8. . 14400 10.1126/science.14400

[25] Perluigi M, Swomley AM, Butterfield DA. Redox proteomics and the dynamic molecular landscape of the aging brain. Ageing research reviews. 2014;13:75–89. 10.1016/j.arr.2013.12.005 . 24374232

[26] Calabrese V, Cornelius C, Mancuso C, Lentile R, Stella AM, Butterfield DA. Redox homeostasis and cellular stress response in aging and neurodegeneration. Methods in molecular biology. 2010;610:285–308. 10.1007/978-1-60327-029-8_17 . 20013185

[27] Giordano G, White CC, Costa LG. Assessment of glutathione homeostasis. Methods in molecular biology. 2011;758:205–14. 10.1007/978-1-61779-170-3_14 . 21815068

[28] Zhu Y, Carvey PM, Ling Z. Age-related changes in glutathione and glutathione-related enzymes in rat brain. Brain Res. 2006;1090(1):35–44. 10.1016/j.brainres.2006.03.063 16647047 PMC1868496

[29] Chen TS, Richie JP Jr, Lang CA. The effect of aging on glutathione and cysteine levels in different regions of the mouse brain. Proceedings of the Society for Experimental Biology and Medicine Society for Experimental Biology and Medicine. 1989;190(4):399–402. . 2928355 10.3181/00379727-190-42879

[30] Perluigi M, Di Domenico F, Giorgi A, Schinina ME, Coccia R, Cini C, et al Redox proteomics in aging rat brain: involvement of mitochondrial reduced glutathione status and mitochondrial protein oxidation in the aging process. J Neurosci Res. 2010;88(16):3498–507. 10.1002/jnr.22500 . 20936692

[31] Rebrin I, Kamzalov S, Sohal RS. Effects of age and caloric restriction on glutathione redox state in mice. Free radical biology & medicine. 2003;35(6):626–35. 12957655 10.1016/s0891-5849(03)00388-5 PMC2837076

[32] Zhang C, Rodriguez C, Spaulding J, Aw TY, Feng J. Age-dependent and tissue-related glutathione redox status in a mouse model of Alzheimer's disease. Journal of Alzheimer's disease: JAD. 2012;28(3):655–66. 10.3233/JAD-2011-111244 22045490 PMC4221633

[33] Bak LK, Schousboe A, Waagepetersen HS. The glutamate/GABA-glutamine cycle: aspects of transport, neurotransmitter homeostasis and ammonia transfer. J Neurochem. 2006;98(3):641–53. 10.1111/j.1471-4159.2006.03913.x . 16787421

[34] Yudkoff M. Brain metabolism of branched-chain amino acids. Glia. 1997;21(1):92–8. . 9298851 10.1002/(sici)1098-1136(199709)21:1<92::aid-glia10>3.0.co;2-w

[35] Zwingmann C, Richter-Landsberg C, Brand A, Leibfritz D. NMR spectroscopic study on the metabolic fate of [3-(13)C]alanine in astrocytes, neurons, and cocultures: implications for glia-neuron interactions in neurotransmitter metabolism. Glia. 2000;32(3):286–303. . 11102969 10.1002/1098-1136(200012)32:3<286::aid-glia80>3.0.co;2-p

[36] Zwingmann C, Richter-Landsberg C, Leibfritz D. 13C isotopomer analysis of glucose and alanine metabolism reveals cytosolic pyruvate compartmentation as part of energy metabolism in astrocytes. Glia. 2001;34(3):200–12. . 11329182 10.1002/glia.1054

[37] Suzuki A, Stern SA, Bozdagi O, Huntley GW, Walker RH, Magistretti PJ, et al Astrocyte-neuron lactate transport is required for long-term memory formation. Cell. 2011;144(5):810–23. 10.1016/j.cell.2011.02.018 21376239 PMC3073831

[38] Rathbone MP, Middlemiss PJ, Gysbers JW, Andrew C, Herman MA, Reed JK, et al Trophic effects of purines in neurons and glial cells. Prog Neurobiol. 1999;59(6):663–90. . 10845757 10.1016/s0301-0082(99)00017-9

[39] Chang R, Algird A, Bau C, Rathbone MP, Jiang S. Neuroprotective effects of guanosine on stroke models in vitro and in vivo. Neuroscience letters. 2008;431(2):101–5. 10.1016/j.neulet.2007.11.072 . 18191898

[40] Di Iorio P, Ballerini P, Traversa U, Nicoletti F, D'Alimonte I, Kleywegt S, et al The antiapoptotic effect of guanosine is mediated by the activation of the PI 3-kinase/AKT/PKB pathway in cultured rat astrocytes. Glia. 2004;46(4):356–68. 10.1002/glia.20002 . 15095366

[41] Pettifer KM, Kleywegt S, Bau CJ, Ramsbottom JD, Vertes E, Ciccarelli R, et al Guanosine protects SH-SY5Y cells against beta-amyloid-induced apoptosis. Neuroreport. 2004;15(5):833–6. . 15073525 10.1097/00001756-200404090-00019

[42] Pettifer KM, Jiang S, Bau C, Ballerini P, D'Alimonte I, Werstiuk ES, et al MPP(+)-induced cytotoxicity in neuroblastoma cells: Antagonism and reversal by guanosine. Purinergic signalling. 2007;3(4):399–409. 10.1007/s11302-007-9073-z 18404453 PMC2072917

[43] Li DW, Li GR, Lu Y, Liu ZQ, Chang M, Yao M, et al alpha-lipoic acid protects dopaminergic neurons against MPP+-induced apoptosis by attenuating reactive oxygen species formation. International journal of molecular medicine. 2013;32(1):108–14. 10.3892/ijmm.2013.1361 . 23615851

[44] Lopez M, Vidal-Puig A. Brain lipogenesis and regulation of energy metabolism. Current opinion in clinical nutrition and metabolic care. 2008;11(4):483–90. 10.1097/MCO.0b013e328302f3d8 . 18542011

[45] Schlesinger I, Schlesinger N. Uric acid in Parkinson's disease. Mov Disord. 2008;23(12):1653–7. 10.1002/mds.22139 . 18618666

