# Supplementary figures and images for: Longitudinal Metabolomics Profiling of Parkinson’s Disease-Related α-Synuclein A53T Transgenic Mice
Source: PLoS One. 2015 Aug 28;10(8):e0136612. doi: 10.1371/journal.pone.0136612 (PMC4552665; doi:10.1371/journal.pone.0136612)

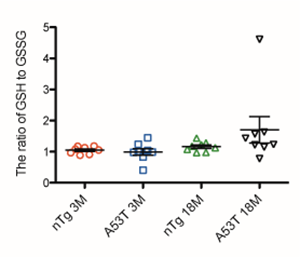

Supplement: S1 Fig — (TIF) [file pone.0136612.s002.tif]
